# Gastric wall implantation metastasis of retroperitoneal extraskeletal osteosarcoma: A case report and review of the literature

**DOI:** 10.3892/ol.2014.2602

**Published:** 2014-10-10

**Authors:** YAN LIU, BIN HU, JING-XIA LI, LU-QI XING, BAO-PING LIU

**Affiliations:** 1 Department of Nuclear Medicine, The First Affiliated Hospital of Zhengzhou University, Zhengzhou, Henan 450052, P.R. China; 2 Department of Nuclear Medicine, The First Affiliated Hospital of HenNan University of Science and Technology, Luoyang, Henan 471003, P.R. China; 3 Department of Oncology, The First Affiliated Hospital of HenNan University of Science and Technology, Luoyang, Henan 471003, P.R. China; 4 Department of Pathology, The First Affiliated Hospital of HenNan University of Science and Technology, Luoyang, Henan 471003, P.R. China

**Keywords:** retroperitoneal extraskeletal osteosarcoma, implantation metastasis, therapy

## Abstract

Retroperitoneal extraskeletal osteosarcoma (ESOS) is a rare and highly invasive tumor that is usually diagnosed at an advanced stage due to the insidious onset. The present study analyses a case of retroperitoneal ESOS and its clinical, radiological and therapeutic conditions, and also provides a review of the literature. A 52-year-old male was diagnosed with retroperitoneal ESOS. The patient succumbed to the condition one year after the initial surgery. During treatment, the patient underwent two additional surgeries and two courses of chemotherapy. In the present case, a peritoneal metastatic lesion of ESOS was shed from the peritoneum and implanted into the outer membrane of the stomach and metastasis was identified, this has rarely been reported in the literature. Retroperitoneal ESOS should be considered in the differential diagnosis of a retroperitoneal mass in order to facilitate the management of surgery and help determine the appropriate treatment of the disease.

## Introduction

Extraskeletal osteosarcoma (ESOS) is a rare and highly invasive tumor (1–3,5–8). Retroperitoneal ESOS is usually diagnosed at an advanced stage due to the insidious onset (2–9). The lungs and liver are the most common sites of metastases (1,7–10). The present study reports a case of gastric parietal implantation metastasis and peritoneum multiple metastases on retroperitoneal ESOS. The literature on retroperitoneal ESOS is also reviewed.

## Case report

**Figure 1:**
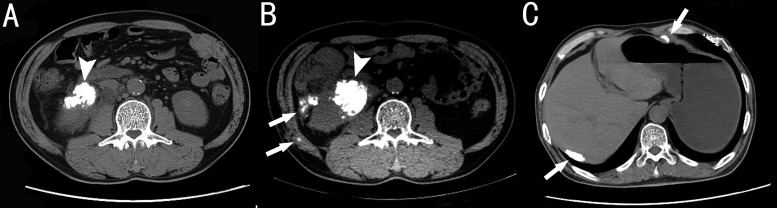
Representative abdominal computed tomography (CT) findings (A) prior to and (B and C) following the first surgery. (A) Prior to the first surgery, abdominal CT showed a large dense mass with calcification (arrowhead) located below the right kidney. (B) Two months after the first surgery, CT demonstrated a mass (arrowhead), with a large calcified lesion, anterior to the right psoas muscle. The renal fascia and abdominal wall showed multiple calcified foci (arrow). (C) Nine months after the first surgery, CT revealed calcified lesions (arrow) in the outer stomach anterior wall and hepatic surface.

**Figure 2:**
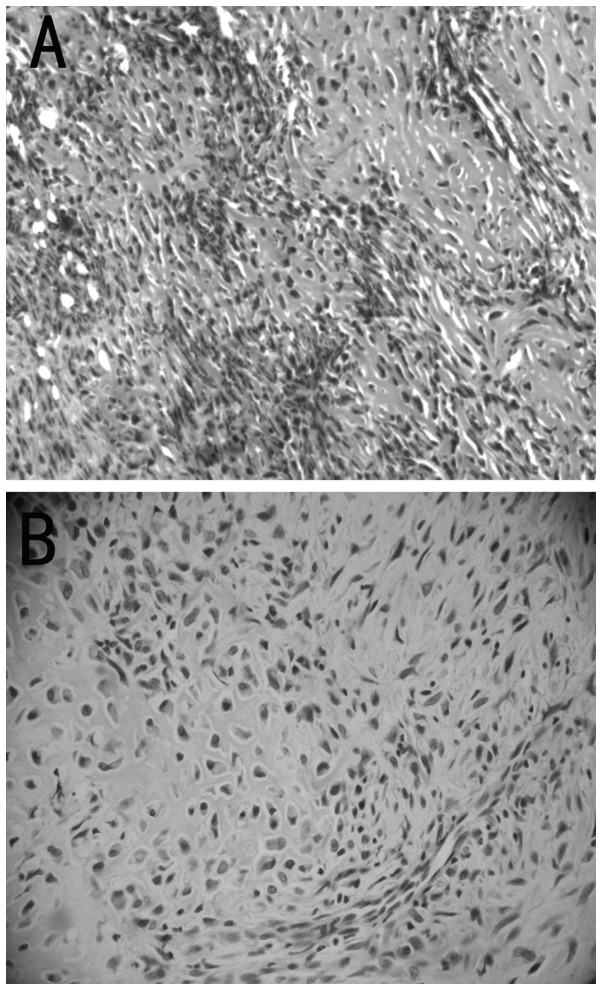
Representative images of hematoxylin and eosin staining. (A) Histological appearance of the first surgical mass specimen. The tumor was composed of spindle- and polygonal-shaped tumor cells, with a banded or irregular osteoid matrix. The tumor cells exhibited varying degrees of atypia, and visible mitotic figures were present (magnification, ×200). (B) Histological appearance of the third surgical gastric specimen. Osteosarcoma components were visible in the outer membrane of the stomach wall (magnification, ×400).

A 52-year-old male was hospitalized with intermittent pain in the right abdomen that had persisted for one week. The medical history revealed hypertension, but no history of trauma and radiation exposure or a family history of genetic diseases. Physical examination showed a large, hard, immobile mass with a smooth surface, ~6×6 cm in size. Laboratory tests revealed a small increase in the serum creatinine level to 120 μmol/l (normal range, 40–110 μmol/l), while the remaining results, including that for alkaline phosphatase (ALP), were normal. An abdominal computed tomography (CT) scan (Fig. 1) showed a large, dense mass, with calcification, located below the right kidney, an oppressed upper ureter and thickening of the renal fascia. An exploratory laparotomy discovered a stiff calcified immobile retroperitoneal mass of 5×6 cm, with a wide base below the right kidney. The mass could not be completely resected of its attachment to the surrounding organs. Pathology revealed that the tumor was composed of spindle- and polygonal-shaped tumor cells, with a banded or irregular osteoid matrix. The tumor cells exhibited varying degrees of atypia and visible mitotic figures (Fig. 2). From these results, a diagnosis of extraskeletal osteosarcoma was formed. Immunohistochemistry showed the positive expression of vimentin and S-100, whereas examination of cytokeratin, cluster of differentiation (CD)117, CD34, epithelial membrane antigen, melanoma, B-cell lymphoma-2 and CD99 staining was negative.

Two months after the surgery, CT imaging (Fig. 1B) revealed a retroperitoneal ESOS near the right upper ureter, with a large amount of calcification. The imaging also revealed multiple metastases of the hepatic capsular, renal fascia and peritoneum. After four months, the patient underwent a second exploratory laparotomy due to tumor relapse. The surgery demonstrated that the retroperitoneal mass of ~10×10 cm in size was closely adhered to the right kidney, ileocecum and the posterior abdominal wall. There was significant chondroid tissue present on the omental tumors, which were 0.5×1.5 cm in size. Only the omental metastases were cut, and cytoreductive surgery was not viable due to the multiple metastases of the abdominal cavity and the severe adhesion with the retroperitoneal tissue. Pathological examination showed that the tumor tissue was predominantly composed of spindle-shaped cells and differentiated immature bone tissue. The cells showed mild-moderate atypia and were ordered in a storiform arrangement, with visible mitotic figures. From these results, a diagnosis of an omental osteosarcoma was formed.

The patient was administered two courses of chemotherapy, where each cycle lasted 28 days. During each cycle Endostar (15 mg/day for the first 14 days), cisplatin (100 mg/m^2^ on the first day) and epirubicin (25 mg/m^2^ for the first three days) were administered intravenously, but this had minimal efficacy. Five months after the second surgery, the patient experienced vomiting and an incomplete intestinal obstruction was suspected. The patient therefore underwent a third exploratory laparotomy. The surgery revealed a 25×30-cm right retroperitoneal mass oppressing the descending section of the duodenum and surrounded the descending colon, and a 1.1×1.2-cm implantation metastasis nodule in the outer membrane of the gastric body anterior wall (Fig. 1C). The liver and spleen exhibited no metastatic nodules. A gastrojejunostomy and ileocolonic anastomosis were performed, and the pathology (Fig. 2B) revealed components of an osteosarcoma in the outer stomach wall, in accordance with a gastric wall osteosarcoma metastasis. The serum ALP level gradually increased to 199 U/l (normal range, 40–130 U/l). The patient eventually succumbed to retroperitoneal ESOS one year after the first surgery.

## Discussion

ESOS represents <4% of all osteosarcomas and 1–2% of all soft-tissue sarcomas (1–3). There is a male bias for osteosarcoma, and the gender ratio is 1.9:1.0 (2). The most common sites of ESOS are the soft tissues of the limbs and the retroperitoneum (11). Retroperitoneal ESOS is a typical osteosarcoma, identified in the retroperitoneal soft tissue with no attachment to the bones or bone periosteum, and producing osteoid or cartilage matrix (12). The incidence rate of retroperitoneum ESOS accounts for 17% of ESOS (8,13). ESOS occurs predominantly in elderly individuals over 50 years old, which differs from osteosarcoma (14). In total, 10 cases of retroperitoneal ESOS, including the present case, have been reported in the literature (1–9) (Table II); these included five males and five females at a gender ratio of 1:1. The average ages of the male and female cases are 64.8 and 68.6 years respectively, with a range of 52–80 years. Five tumors (50%) occurred in the right abdomen, three in the left abdomen (30%), one in the pelvis (10%) and one tumor location was unavailable. Three cases presented with hydronephrosis or hydroureterosis due to tumor compression. All 10 patients exhibited calcification to varying degrees, which facilitated diagnosing the disease. The minimum diameter of the tumors was >5 cm. Nine cases (90%) occurred with surrounding invasive or distant metastases, and one case was not mentioned. Eight cases (80%) were treated with surgery combined with chemotherapy, including one case with interventional therapy, and the treatments of two cases were unavailable. None of these treatment options improved the survival rate.

A total of 93% of cases of retroperitoneal ESOS showed increasing soft-tissue masses with insidious onset, and 65–80% of patients experienced pain (2). The onset of retroperitoneal ESOS is commonly asymptomatic due to the large lacuna volume of the retroperitoneum, which provides sufficient space for tumor growth. In the present case, once the disease had progressed to a certain stage, the patient experienced discomfort in the abdomen from the tumor oppression to the surrounding tissue. In this case, the patient was not hospitalized until there was discomfort to the urinary system, caused by the tumor oppression to the right ureter. Retroperitoneal ESOS is peculiarly prone to recurrence and metastasis, as the tumor often invades the surrounding vital organs, making it difficult to completely excise the mass. There is no specific tumor marker for the auxiliary diagnosis of ESOS. However, Narayanan (15) found that in ESOS, the ALP level was often increased, which was established as a prognostic factor. The serum ALP level of this patient was normal at the onset of the ESOS, but rose gradually with the progression of the disease.

Retroperitoneal ESOS is usually discovered by imaging, which identifies a homogeneous soft-tissue mass with calcification. A calcified retroperitoneal mass may have a wide variety of differential diagnoses, which include several benign and malignant conditions (1,16–20). Malignant lesions include malignant fibrous histiocytomas, malignant stromal tumors and extraskeletal chondrosarcomas, and the differential diagnoses for these are commonly based on histopathology. In the present case, the histopathology of the primary retroperitoneal tumor, omentum and gastric metastases all revealed that the patient was suffering from a retroperitoneal ESOS, however, the data from the histological assessment showed large variation. Therefore, the diagnosis of ESOS should be based on a combination of clinical, radiographical and pathological findings (10). X-ray of the ESOS showed a soft-tissue mass with or without calcification, while CT characteristic imaging revealed a calcified, high-density mass. Magnetic resonance imaging of the calcification and bone tumors of ESOS is not as informative as CT, but it is superior to CT in identifying the tissue components and determining the association between the tumor boundaries and the surrounding tissue (21). Enhanced CT manifestations of ESOS can be diversiform (13), hypervascular or poorly vascular. As ESOS exhibits a variety of histological manifestations, CT-guided biopsy is recommended only when lymphoma or germ cell tumors are suspected (22). The diagnoses of the majority of patients are therefore confirmed based on the pathological findings during or following surgery.

The comprehensive treatment of ESOS is based on surgical intervention, and the effects of chemotherapy and radiotherapy are poor. There has been one previous study (3) on an interventional surgery for ESOS, however, the effects require further evaluation. The main treatment for retroperitoneal ESOS is surgery, but caution is recommended since the volume of the tumor may be too large to be removed completely. It has been reported that improved survival can be observed following radical resection and wide excision at the time of the first surgery (4,23). Lee *et al* (24) identified that more aggressive surgical treatment for recurrence was useful for local control, but did not decrease the incidence of mortality due to the disease. Therefore the advantages and disadvantages of pre-operative and intraoperative should be evaluated. Premature surgery will increase the rate of the transfer, which could be otherwise avoided. As in the present case, the possibility of malignancy should be taken into account in retroperitoneal tumors due to the attachment to the surrounding tissue. A more appropriate surgical approach could be applied, such as a kidney ventriculostomy instead of cytoreductive surgery, to relieve hydronephrosis. In the present case, the surgery could not remove the tumor completely or prolong the life of the patient. This may have also increased the possibility of metastasis. When the patient underwent the second surgery, there was no medical value in resecting the primary tumor, therefore, only the omental lesions were removed. The patient was administered chemotherapy, but the effects were unsatisfactory. Five months after the second surgery, the patients experienced continual vomiting and underwent a third exploratory laparotomy due to an incomplete intestinal obstruction. The surgeries included a gastrojejunostomy and ileocolonic anastomosis, and cytoreductive surgery was not performed. It is important to consider retroperitoneal ESOS in the differential diagnosis of a retroperitoneal mass in order to guide the management of surgery and determine the most effective treatment for the disease.

The five-year survival rate for patients with ESOS is <37% (4,25). The volume of the tumor is an important factor in the prognosis of ESOS, a volume >5 cm is usually associated with a poor prognosis (10). In the present case, complete removal of the retroperitoneal tumor was difficult, as the volume was too large, therefore, the prognosis was extremely poor. The most common metastases of ESOS occur in the lung and liver (1,7–10). However, in the present case, a gastric wall implantation metastasis was identified, which has rarely been reported in the literature.

## Figures and Tables

**Table I:** Details of the three surgeries performed on the patient with retroperitoneal ESOS.

Surgical details	First surgery	Second surgery	Third surgery
Date of surgery	March 06, 2012	July 18, 2012	Dec 24, 2012
Pre-operative diagnosis	Right hydronephrosis	Right abdominal tumor recurrence	Incomplete intestinal obstruction
Surgical aim	Surgery on right kidney hydronephrosis	Cytoreductive surgery	Relief of intestinal obstruction
Intraoperative findings	Retroperitoneal mass below the right kidney and oppressing the ureter	Retroperitoneal mass closely adherent to the right kidney, ileocecum and posterior abdominal wall	Right retroperitoneal mass oppressing the descending section of the duodenum and surrounding the descending colon
Tumor size, cm^2^	8v8	10×12	25×30
Gross appearance	A stiff, calcified, immobile retroperitoneal mass with a wide base	A stiff, calcified, immobile retroperitoneal mass with a wide base	A stiff, calcified, immobile retroperitoneal mass with a wide base
Relapse or distant transfer of disease	Non-metastasis	Intraperitoneal and abdominal wall metastases	New metastasis of the gastric wall outer membrane
Intraoperative treatment	Partial resection of right peritoneal tumor	Only omental metastases were cut	Gastrojejunostomy and ileocolonic anastomosis
Post-operative pathology	Retroperitoneal ESOS	Omental osteosarcoma metastasis	Gastric wall outer membrane osteosarcoma metastasis

ESOS, extraskeletal osteosarcoma.

**Table II:** Reported cases of retroperitoneal ESOS.

Case no.	Gender/age, years	Location	Symptoms of tumor compression	Tumor calcification	Tumor size, cm	Surrounding invasion	Therapy	Reference no.
1	Female/66	Right iliac fossa	Bilateral hydronephrosis	Calcification	NA	Invasion	Chemotherapy	(1)
2	Female/67	Inferior left renal	Left Ureter Obstruction	Calcification	17	Invasion	Surgery	(2)
3	Female/74	Above left renal	Null	Calcification	16	Invasion	Interventional therapy and chemotherapy	(3)
4	Female/74	Right renal region	Null	Calcification	NA	Invasion	N/A	(4)
5	Male/80	Peripheral right renal	N/A	Calcification	10	Invasion	Surgery	(5)
6	Female/62	Rright renal region	Null	Calcification	14	Invasion	Surgery	(6)
7	Male/58	Anterior left renal	Null	Calcification	6.5	Invasion	Chemotherapy	(7)
8	Male/68	Inferior right renal	N/A	Calcification	19	NA	N/A	(8)
9	Male/66	NA	N/A	Calcification	NA	Invasion	Surgery	(9)
10	Male/52	Inferior right renal	Right kidney hydronephrosis	Calcification	6	Invasion	Surgery and chemotherapy	Present case

NA, not available; ESOS, extraskeletal osteosarcoma.

## Abbreviations

ESOS: extraskeletal osteosarcoma
ALP: alkaline phosphatase
CD: cluster of differentiation
CT: computed tomography
